# Size-Dependent Tissue Translocation and Physiological Responses to Dietary Polystyrene Microplastics in *Salmo trutta*

**DOI:** 10.3390/ani16020285

**Published:** 2026-01-16

**Authors:** Buumba Hampuwo, Anna Duenser, Elias Lahnsteiner, Thomas Friedrich, Franz Lahnsteiner

**Affiliations:** 1 Fishfarm Kreuzstein, Oberburgau 28, 4866 Unterach, Austria; 2 Institute of Hydrobiology and Aquatic Ecosystem Management, Boku University, Gregor-Mendel-Straße 33, 1180 Wien, Austria; 3 Federal Agency for Water Management, Institute for Water Ecology, Fisheries and Lake Research, Scharfling 18, 5310 Mondsee, Austria

**Keywords:** microplastics, polystyrene, bioaccumulation, *Salmo trutta*, enzyme activities, fluorescence microscopy

## Abstract

Microplastics (MPs) are prevalent in freshwater systems; consequently, fish ingest them either accidentally or intentionally. Once ingested, MPs can translocate to various organs and cause physiological effects. Most studies have focused on tropical and marine fishes, and many have used mass-based methods that measure exposure only by the total mass of microplastics, ignoring particle number and size. These studies have also rarely examined MP effects or fate after a depuration period, limiting our understanding of MP impacts on temperate fishes, hindering the harmonisation of toxicological studies, and complicating assessments of food safety for cultured and wild fish. This study investigated the physiological impacts of dietary exposure to polystyrene microplastics (PS-MPs; 1–10 µm) in *Salmo trutta* fed a diet with ~5.4 × 10^6^ PS-MPs g^−1^ feed for 21 days, followed by a 90-day depuration period. PS-MPs translocation from the intestine to the liver and muscle was investigated. Enzymatic biomarkers of oxidative stress and metabolism were analysed in the liver, digestive enzyme activity was assessed in the intestine, and inflammatory enzyme responses were evaluated in both liver and intestinal tissues. In addition, malondialdehyde (MDA) concentration, an indicator of lipid peroxidation, was quantified in blood, muscle, and liver samples. Results show that 1–5 µm PS-MPs translocated to the liver and muscle, while 10 µm particles largely remained in the intestine, with a small fraction detected in muscle tissue but not in the liver. Most biochemical markers were unaffected; however, both trypsin and peroxidase activities significantly decreased after 21 days, and lipid peroxidation increased in blood following 90 days of depuration. PS-MPs persisted in muscle following 90 days of depuration. These findings demonstrate that dietary exposure to PS-MPs in the size range 1–10 µm leads to selective physiological alterations in *S. trutta* and results in persistent accumulation of MPs in organs, especially muscle tissue consumed by humans, highlighting a clear concern for food safety.

## Simple Summary

Microplastics are widespread pollutants found in freshwater, glaciers, and even the air. This study examined how polystyrene microplastics (PS-MPs; 1–10 µm) affect brown trout, a temperate freshwater fish important for ecology and aquaculture. Fish were fed polystyrene microplastic-spiked diets of approximately 5 million MPs per gram of feed for 21 days, followed by 90 days on clean feed. Small PS-MPs (1–5 µm) moved from the gut to the liver and muscle, while larger MPs (10 µm) mostly stayed in the intestine, with a small fraction reaching muscle tissue. Most biochemical markers were unaffected, but some enzymes, including trypsin, which is responsible for protein digestion, and peroxidase, which helps remove reactive oxygen species, were significantly decreased after 21 days. Importantly, MPs persisted in muscle tissue even after depuration, representing a clear concern for human food safety.

## 1. Introduction

Microplastics (MPs), which were thought to be primarily prevalent in marine systems, have become ubiquitous in the environment. They are now being detected in freshwater ecosystems, including lakes, rivers, and sediments [1,2,3]. MPs have also been found in unexpected places, including high-altitude remote lakes [4], Himalayan glaciers [5], and even household air [6,7], making them one of the most common pollutants of the twenty-first century. Consequently, research aimed at understanding the fate and effects of MPs and their additives on organisms has increased substantially in recent years. Many of these studies have focused on aquatic species, particularly fish [8], as fish ingest MPs either accidentally or intentionally. MPs can subsequently translocate to various organs, including muscle tissue consumed by humans [9]. Once located in the organs and cells, MPs may cause inflammation, oxidative stress, disruption of cell signalling and cellular biochemical processes, hormonal disruptions, immune system suppression, genetic damage, and reduction in fish growth and reproduction rates [10,11].

Several organ-related effects have been reported across different fish species exposed to microplastics. For example, in zebrafish (*Danio rerio*), exposure to a mix of PS-MPs and high-density polyethene MPs (100 µg L^−1^ and 1000 µg L^−1^) induced extensive transcriptional alterations in the head kidney, even at the lower exposure concentration [12]. In crucian carp (*Carassius carassius*), PS-MPs in a size range of 50–500 µm accumulated predominantly in the intestinal tract and gills, resulting in histopathological damage to these tissues and altered gene expression in the liver, intestine, and gills [13]. Goldfish (*Carassius auratus*) exposed to virgin MPs of varying sizes and morphologies (fibres, fragments, and pellets) exhibited pronounced hepatic histological alterations accompanied by inflammatory responses [14]. In contrast, medaka (*Oryzias latipes*) exposed to heterogeneous virgin PS-MPs with an average size of 200 µm at concentrations of 50 and 500 µg L^−1^ showed only minor histological alterations and a temporary delay in reproduction, which normalised over the course of the experiment [15]. Likewise, African catfish *(Clarias gariepinus*) exposed to 50 or 500 µg L^−1^ low-density polyethene preloaded with phenanthrene exhibited histological damage in liver and gill tissues, along with transcriptional changes in several target genes [16]. Microplastics have also been reported to affect haematological parameters, including reductions in red blood cells, white blood cells, and haemoglobin concentrations in Indian carp (*Catla catla*) [17], rohu carp (*Labeo rohita*) [18], and mrigal carp (*Cirrhinus mrigala*) [19], and brown trout (*Salmo trutta*) [20].

From the literature reviewed above, it is evident that most studies have focused on marine and tropical fish species, whereas studies on temperate freshwater fish are limited, particularly on salmonids. This limitation has historically assumed that temperate systems contain negligible amounts of MPs compared with marine and tropical environments. However, increasing evidence indicates that microplastic concentrations in temperate freshwater systems in Europe are rising, particularly due to tyre wear [21,22,23].

Existing studies on salmonids have primarily examined Atlantic salmon (*Salmo salar*) and rainbow trout (*Oncorhynchus mykiss*) and have reported molecular, proteomic, and physiological responses following exposure to various polymer types and particle sizes [24,25,26]. Notably, the majority of studies reviewed above employed microplastic size classes larger than 20 µm and used mass-based dosages. This is problematic because particles greater than 20 µm rarely cross the intestinal barrier or localise within cells [19,27,28], and mass-based toxicological exposure does not adequately address the contribution of small MPs to total mass.

This study investigated the effects of dietary exposure of *S. trutta* to 1, 5, and 10 µm PS-MPs on enzyme activities and biochemical markers related to oxidative stress, oxidative damage, inflammatory response, digestion, and metabolism, as well as on PS-MP translocation to the liver and muscle, using a concentration of approximately 5.4 × 10^6^ particles per gram of feed. These endpoints have been examined in other species [12,13,14,15,16,17,18,19,20] but rarely in *S. trutta*. *S. trutta* was selected for the investigations because of its substantial social and economic importance and because of its sensitivity to environmental pollution [29]. PS-MPs were tested because they are among the most abundant polymers in aquatic ecosystems [30,31,32], and dietary exposure was selected as it is the primary natural ingestion route.

Effects of PS-MPs were assessed after 21 days of exposure and following a 90-day depuration period. These sampling points were selected to determine (a) the immediate effects of PS-MPs, (b) whether any effects persist after 90 days of depuration or emerge at this time point, and (c) microplastic concentrations in the intestine, liver, and muscle. We hypothesised that dietary exposure to PS-MPs in the size range of 1–10 µm would result in size-dependent tissue distribution, with smaller particles (1–5 µm) translocating from the intestine to the liver and muscle. We further expected that exposure to the tested PS-MPs concentration would result in physiological disruption, specifically the investigated biomarkers related to oxidative stress, inflammation, digestion, and metabolism. Finally, we hypothesised that MP concentration would be highest on day 21, immediately after exposure, and systematically reduce following 90 days of depuration in the investigated organs.

## 2. Materials and Methods

### 2.1. Fish and Experimental Design

*S. trutta* used in the experiment were obtained from the Kreuzstein fish farm. Initially, the fish were kept in a single communal circular tank under standard aquaculture flow-through conditions. From this tank, 160 fish (average weight: 31 ± 8 g; total length: 14 ± 2 cm) were randomly selected and evenly distributed into four rectangular flow-through stream channels measuring 1.5 × 0.25 × 0.35 m (length × width × height). The resulting biomass was 40 fish (1.25 kg) per tank. The tanks were supplied with groundwater with a temperature of 9.9 ± 0.3 °C and a flow rate of 0.15 L s^−1^. Two stream channels served as controls, while the other two were used for treatment. Flow-through conditions were used in the experiments. These are advantageous, as constant water conditions can be maintained throughout the experiment and tank effects are very unlikely. The experiment employed very low stocking densities, which are not representative of typical aquaculture conditions, where at least fivefold higher stocking densities are commonly used. This was due to several reasons. First, the number of experimental fish was limited by the animal experiment permit for ethical reasons. Second, the study aimed to investigate the responses of individual fish to MP exposure, as fish may differ substantially in physiology, metabolic rate, and overall condition. Consequently, each fish was treated as an individual experimental unit, and tank replicates were included only as a precautionary measure rather than as experimental replicates.

### 2.2. Ethical Statement

The study is a registered animal experiment of the Austrian Ministry of Education, Science and Research (permit number: 2024—0.826.292). Obligations under this permit include compliance with Austrian regulations governing animal welfare and protection, as well as the EU Directive 2010/63/EU for animal experiments. All practices and procedures for the care and management of animals were performed under current best practice and supervised by experienced scientists and a veterinarian.

### 2.3. Microplastic Concentration and Feed Preparation

Aqueous suspensions of spherical PS-MPs with a diameter of 1 µm, 5 µm and 10 µm and with defined concentrations were acquired from Sigma-Aldrich, Darmstadt, Germany. PS-MPs were incorporated into commercial trout feed. Firstly, the feed was ground, and the stock solutions were diluted to the desired final particle number using the dilution formula *C*_1_ *× V*_1_ *= C*_2_ *× V*_2_ (*C*_1_ = stock concentration of MPs, *V*_1_ = stock volume, *C*_2_ = desired concentration in feed, and *V*_2_ = desired volume after dilution), after ensuring homogeneous resuspension of MPs in the stock by vortexing and shaking while pipetting. Then the diluted PS-MPs suspension was slowly poured into the ground feed in circular motion, maintaining a 1:5 ratio (microplastic suspension: ground feed, *w*/*w*). The components were mixed thoroughly to ensure a homogeneous distribution of microplastics, resulting in a dough that was then extruded using a Caleva bench-top extruder (Caleva Process Solutions Ltd., Sturminster Newton, Dorset, England). The extrudate was processed into 1.5 mm pellets suitable for the experimental fish using a Caleva spheronizer extruder (Caleva Process Solutions Ltd., Sturminster Newton, Dorset, England) and dried at 70 °C for 12 h. Then the control feed was prepared using the same procedure, except no microplastic was added. To prevent residual contamination of MPs from the machine in the control feed, the control feed was prepared first. Confirmation of PS-MPs in the feed was also performed analytically (see Section 2.6).

The current study uses high particle numbers (Table 1) compared to reports in environmental assessments. 25.8 MP particles L^−1^ water were detected in Taihu Lake, China, 100 particles mL^−1^ in the Amsterdam canals in the Netherlands, and up to 3605 particles mL^−1^ in the Czech Republic, as cited in [33]. When MP concentration was not expressed in particle numbers but as mass, 229–1182 µg MP L^−1^ water were found in Taihu Lake, China [34], 790–1560 µg L^−1^ (size range 53–105 µm) in a lake in Texas, USA [35], and 714 µg L^−1^ (size range 10–300 µm) in the Rhine River in Germany [36]. In aquaculture, reported burdens in farmed salmonids are around ∼1.8 × 10^3^–9.4 × 10^3^ MPs per fish throughout the entire culture period [37,38]. However, all these reports exclude particles < 20 µm because of analytical limits, though small MPs < 20 µm may be more abundant in the environment. For this reason, the present study deliberately employed high concentrations of PS-MPs in the 1–10 μm size range. Although environmental concentrations of particles in this size class are not well constrained, these smaller MPs are considered more biologically mobile and more likely to translocate to internal organs following ingestion [9]. Additionally, the abundance of small MPs may be underestimated, as they contribute relatively little to the total MP mass compared with larger particles. The mass-converted concentrations applied in this study fall within the range of mass-based concentrations used in previous studies on *S. salar* (0.002 µg MP g^−1^ to 200 µg MP g^−1^ feed [24]) and *O. mykiss* (500–1000 µg MP g^−1^ [39]). It also falls in the range detected in various human tissues (5–500 µg MP g^−1^ tissue [40,41,42,43,44]).

### 2.4. Fish Exposure

Fish were acclimatised to the rearing system for five days before the start of the experiment. Feed was administered using band feeders for 12 h daily over a 21-day exposure period. The daily ration was set to be completely consumed, resulting in a feeding ratio of 0.8–1% of the fish’s body weight. Following the exposure period, the fish were switched to standard commercial feed without added microplastics and observed for an additional 90 days. The channels were inspected twice daily, in the morning and evening, for mortalities, behavioural abnormalities, and symptoms of disease. Faecal remnants were removed twice daily in accordance with the fish farm’s hygienic protocols.

### 2.5. Sampling of Fish for the Different Analytical Procedures

Fish were euthanised using MS-222, and body mass and total length were recorded. On day 21 of polystyrene-feed administration, five fish were randomly sampled from each stream channel to assess the immediate effects of microplastics. This sampling point was selected because MPs may distribute to target organs, exert physiological effects, or be eliminated from the organism within this period. Additional sampling was conducted 90 days after the cessation of polystyrene feeding to assess potential long-term effects and to determine whether effects persisted from day 21 or emerged later if previously absent.

For microplastic extraction, 5 fish from each treatment tank were randomly selected (total sample number = 10). The liver, muscle, and intestine were excised, weighed to the nearest gram, and placed in 10% KOH. Samples were stored at room temperature until the start of the microplastic extraction process described in Section 2.6.

For enzymatic activity analyses, 5 fish from each treatment tank were randomly selected (Total n = 10). The liver and intestine were excised, placed in 0.1 mmol L^−1^ Tris buffer pH 7.4 supplemented with 0.1% Triton X100 (Sigma Aldrich, Steinheim, Germany), and stored at −20 °C until analysis. Enzymes related to oxidative stress, digestion, metabolism, and inflammation were quantified. Oxidative stress enzymes included catalase (CAT), peroxidase (POD), and superoxide dismutase (SOD). Digestive enzymes included lipase, trypsin, phospholipase, and leucine aminopeptidase. Metabolic enzymes included malate dehydrogenase (MDH), lactate dehydrogenase (LDH), and pyruvate kinase (PK). The inflammatory enzyme caspase-1 was also analysed. Enzyme activities were measured in the liver, intestine, or both, depending on their biological relevance. Additionally, the oxidative damage marker, malondialdehyde (MDA), assessed by lipid peroxidation (thiobarbituric acid reactive substances-TBARS assay), was measured in the liver, muscle, and blood. Blood was collected via the heart ventricle. Tissues were collected as described above. Tissue and blood samples were weighed and immediately stored in liquid nitrogen (−196 °C) until analysis.

### 2.6. Microplastic Extraction and Detection

Microplastic extraction, detection, and quantification were performed using a slightly modified method as used by Lahnsteiner et al. [20]. Briefly, liver, muscle and intestine were dissected from 10 fish. Tissue samples and feed samples were weighed, then digested in 10% (*w*/*v*) potassium hydroxide containing 1% (*v*/*v*) liquid dishwashing detergent at 60 °C for 24 h, using a tissue-to-solution ratio of 1:10. After digestion, samples were cooled to room temperature, and sodium chloride was added until saturation (~35% *w*/*v*). The mixture was transferred to a separation funnel and allowed to settle for 45 min at room temperature, after which the upper 20% of the total volume was collected by discharging the lower phase. The collected phase was mixed with absolute ethanol at a 1:1 ratio; for lipid-rich samples, the sample-to-ethanol ratio was increased to 1:5. If precipitation occurred, distilled water was added until the precipitate dissolved completely. The solution was then vacuum filtered through a cellulose acetate membrane filter (0.45 µm pore size). The filter was cleaned by repeatedly rinsing it with small alternating amounts of ethanol and water (totalling 10 mL each) to completely wash off both lipid and precipitate residues. For microplastic collection, the filter was inverted in the filtration apparatus so that the retained particle side faced downward into a clean Falcon tube, soaked with 2 mL of distilled water for five minutes, and vacuum pressure was applied while 1 mL of water was pipetted across the surface twice; an additional 1 mL of distilled water was used to rinse the filter into the same tube to ensure complete MP transfer.

PS-MPs were stained with iDye Pink 1% (*w*/*v*) (Jaquard Products Healsburg, CA, USA) at room temperature. Particle detection was performed using a Zeiss Axioscope 5 fluorescence microscope (Carl Zeiss, Jena, Germany) with a Neubauer improved counting chamber (10 µm depth), at 400× magnification. The chamber was rinsed three times with 96% ethanol and distilled water to avoid contamination with MP. Then the sample was loaded, and 15 micrographs were taken using an excitation wavelength of 555 nm and a bandwidth of 30 nm.

### 2.7. Microplastic Quantification by Image Analysis

Fluorescence micrographs were analysed using ImageJ software (version 1.54f; National Institutes of Health, Bethesda, MD, USA; https://imagej.net/ij/, accessed on 10 November 2025). The spatial scale for the 40× objective was calibrated using a micrometre calibration slide (Motic Europe S.L.U., Barcelona, Spain). Measurements of polystyrene (PS) particles in the stock suspensions confirmed the nominal diameters of 1 µm, 5 µm, and 10 µm indicated by the manufacturer. Fluorescent particles detected in the samples were classified as PS microplastics (PS-MPs) only if they exhibited a perfectly spherical morphology and a Feret’s diameter of exactly 1 µm, 5 µm, or 10 µm. The total particle number per sample was extrapolated from the mean particle count per microscopic field to the total filtered sample volume. For mass estimation, the volume of individual particles was calculated assuming spherical geometry (V = 4/3 πr^3^). Particle mass was then determined using the equation: mass = polymer density × volume, with a density of 1.05 g cm^−3^. Total MP mass per sample was calculated by multiplying the mass of a single particle by the total number of particles detected in the sample. By restricting the analysis to perfectly spherical particles, the method ensured high specificity for the experimentally introduced PS microplastics (PS-MPs). PS-MPs exhibit high chemical stability and are not susceptible to mechanical fragmentation during ingestion, enzymatic digestion in the intestine, or intracellular lysis [45,46,47]. Consequently, their morphology is not expected to change within the organism. The reported values, therefore, represent a robust, semi-quantitative measure of intact PS-MP translocation, suitable for correlation with biochemical responses.

**Figure 1:**
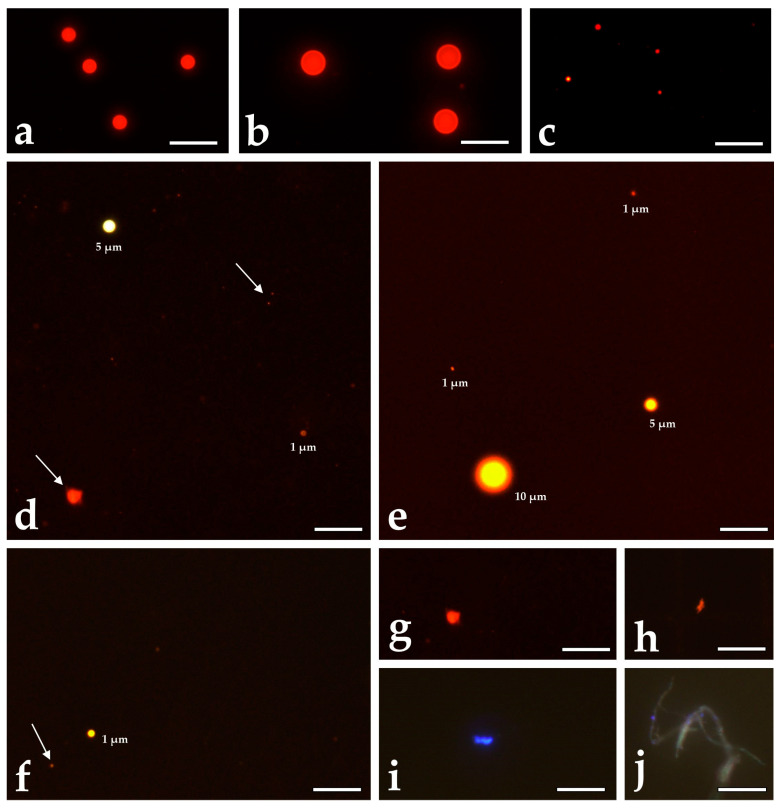
Micrographs of MP spheres used for the experiments and of MP extracted from *S. trutta tissues*. (**a**–**c**): 5 µm, 10 µm, and 1 µm polystyrene spheres from the diluted stock solutions. (**d**–**f**): Microplastic extracted from muscle, intestine and liver. Arrows indicate foreign microplastics. (**g**–**i**): Types of foreign MP detected in tissue. (**j**) sample contamination with a fibre (not included in analysis). Colour intensity of spiked PS-MP depends on focus and light intensity of the microscope; the colour of foreign MP is type-specific. Scale bar = 20 µm.

Particles exhibiting fluorescence after staining with Pink iDye but displaying different diameters or non-spherical morphologies (e.g., irregular, cylindrical, or ellipsoidal structures; Figure 1) were categorised as background contamination from foreign microplastics. These data are reported separately from the PS-MP results (Supplementary File, Table S2). Background concentrations of foreign MPs were quantified only in tissue samples and not in feed. However, because both control and treatment diets were prepared from the same batch of commercial feed and processed identically, background contamination is expected to be uniform across dietary treatments.

Blank procedural controls, in which the KOH digestion solution and filtration setup were processed identically but without tissue, confirmed the absence of spherical particles matching the morphology of the spiked PS-MPs.

### 2.8. Enzymatic Assays and MDA Determination

All enzyme activities were measured at 21 ± 1 °C. CAT, POD and SOD were assayed colourimetrically. For catalase, the substrate was 5 mmol L^−1^ H_2_O_2_, and the assay was at pH 7.0 [48]. H_2_O_2_ substrate not catabolized by catalase was determined with an ammonium molybdate reaction. Peroxidase was assayed in 0.1 mol L^−1^ phosphate buffer (pH 6.0) using 0.1 mol L^−1^ H_2_O_2_ coupled with 0.2 mmol L^−1^ of the colourimetric probe ABTS (2,2′-azino-bis(3-ethylbenzthiazoline-6-sulfonic acid)) as substrates. Superoxide dismutase activity was determined by the inhibition of cytochrome c (2 µmol L^−1^) reduction by the superoxide radical using a xanthine (50 µmol L^−1^), xanthine oxidase (12 U L^−1^) system at a pH of 7.8.

Digestive enzymes were measured colourimetrically. Lipase activity was assayed using 0.3 mmol L^−1^ 4-nitrophenyl dodecanoate at pH 8.2 [49]. Phospholipase activity was measured with 2 mmol L^−1^ 4-nitro-3-octanoyloxy benzoic acid in an assay containing 10 mmol L^−1^ CaCl_2_ and 100 mmol L^−1^ NaCl (pH 8.0) [49]. Trypsin was assayed colourimetrically following Bergmeyer, 1985 [50] using 1 mmol L^−1^ Nα-benzoyl-L-arginine 4-nitroanilide. Additives were 2 mmol L^−1^ EDTA and 2 mmol L^−1^ cysteine, and the pH of the working solution was 7.5. Leucine aminopeptidase was measured with 1 mmol L^−1^ L-leucine p-nitroanilide at pH 7.2 [51].

The metabolic enzymes MDH, LDH, and PK were assayed UV-spectrophotometrically [50]. For LDH, the substrate was 2 mmol L^−1^ pyruvate, and the pH of the working solution was 7.4. For PK, the substrate was 3 mmol L^−1^ phosphoenolpyruvate (assay pH: 7.6), and for MDH, the substrate was 2 mmol L^−1^ malate (assay pH 9.4). The inflammatory enzyme caspase-1 was assayed in a reaction mixture containing 1 mmol L^−1^ of the chromogenic substrate Ac-Trp-Val-Ala-Asp-pNA, 1 mmol L^−1^ dithiothreitol, and 1 mmol L^−1 1^ mercaptoethanol (pH of 7.5) [52].

The oxidative damage marker malondialdehyde (MDA) was determined using the TBARS assay. Samples (10 controls and 10 treatments) were homogenised in RIPA buffer (0.3025 g Tris in mmol L^−1^, 0.7 g NaCl, in mmol L^−1^ 1% sodium deoxycholate, 0.1% SDS, 0.1% BHT, 1% Triton, pH 7.6). After the addition of 10% TCA, centrifugation, and addition of 0.67% TBA reagent, the mixture was incubated at 95 °C for 60 min, cooled, and the absorbance was measured at 532 nm against a blank solution.

All absorbance readings were taken with a Multiskan^TM^ FC Microplate Photometer (Thermo Fisher Scientific, Waltham, MA, USA), and all the chemicals used were analytical grade produced by Sigma-Aldrich.

### 2.9. Data Analysis

All statistical analyses were conducted in R (v4.3.1). Assumptions of normality (Shapiro–Wilk test) and homogeneity of variance (Levene’s test) were not met; therefore, non-parametric Wilcoxon rank-sum tests were used. Comparisons were performed between the control group (C1) and the PS-MP treatment group (T1) at 21 days post-exposure, and between the control group (C2) and the PS-MP treatment group (T2) after 90 days of depuration (day 111). Because the control groups at the two sampling times differed significantly, each treatment group was compared only with its corresponding time-specific control. Results were considered significant at *p* < 0.05. To assess overall biochemical patterns, variables were standardised (z-score) before principal component analysis (PCA) using the factoextra package. Permutational multivariate analysis of variance (PERMANOVA; adonis2 function, vegan package) with Euclidean distance and 999 permutations evaluated effects of organ, sampling time, treatment, and interactions. Separate PERMANOVAs examined exposure (C1 vs. T1) and depuration (C2 vs. T2) phases. Data are presented as boxplots with n = 10 specimens per group. Absorbance values below the detection limit were set to zero and retained in the analysis. All biochemical parameters are additionally reported in Supplementary Table S1 as mean ± standard deviation. Growth rate data were not statistically analysed due to limited sample size and because they were outside the scope of the study.

## 3. Result

### 3.1. Behavioural and Somatic Condition

Over the course of the experiment, which consisted of 21 days of polystyrene feeding followed by a 90-day depuration period, mortality remained below 5% and was similar between the control fish and the polystyrene-exposed fish. No behavioural differences were observed between polystyrene-exposed and control fish. Likewise, growth rates did not differ between control and treatment groups. The mean daily growth rate was 2.1 ± 0.8% for control fish and 1.9 ± 0.6% for polystyrene-exposed fish.

### 3.2. Microplastic Concentrations

In vitro–administered PS-MPs exhibited a perfectly spherical morphology in the stock solution (Figure 1a–c), which was retained in particles extracted from tissues (Figure 1d–f). In contrast, foreign microplastics displayed heterogeneous morphologies, including elongated and irregular shapes (Figure 1d,f,g,j). These are reported separately in the Supplementary File Table S2. Concentrations of spiked PS-MPs in liver, muscle, and intestine were analysed 21 days after exposure (T1) and after 90 days of depuration (T2). Organ- and size-specific comparisons are summarised in Table 2. In the liver, counts of 1 µm and 5 µm PS-MPs showed no significant change between time points, while 10 µm particles were not detected. Similarly, in muscle, numbers of all three size classes did not significantly change. In contrast, the intestine exhibited a significant decrease in 1 µm PS-MPs, with non-significant declines for the 5 µm and 10 µm fractions.

Table 2 Number of polystyrene microplastic particles in different organs after 21 days of exposure and following 90 days of depuration. Values are presented as mean ± standard deviation (SD) (×10^6^ particles g^−1^ tissue). The per cent difference column indicates the calculated percentage change in PS-MP abundance between the 21-day exposure and the subsequent 90-day depuration period.

### 3.3. Oxidative Stress Marker Enzymes Activity

**Figure 2:**
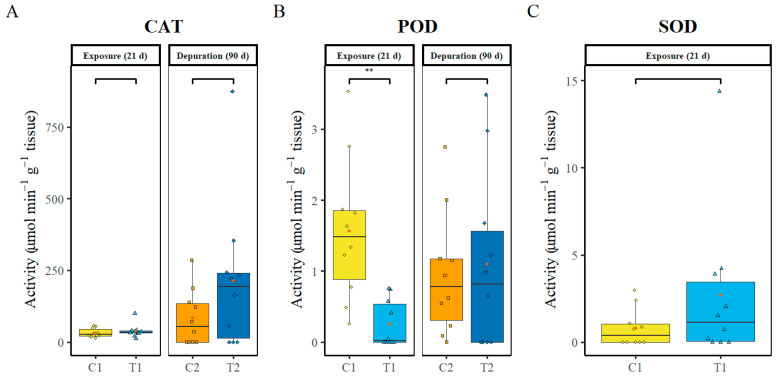
Activities of oxidative stress enzymes in the liver of fish exposed to polystyrene microplastics (**A**) Catalase (CAT), (**B**) Peroxidase (POD), and (**C**) Superoxide dismutase (SOD). C1—control after 21 days, C2—control after 111 days, T1—treatment after 21 days of exposure to PS-MP, T2 treatment after 21 days of exposure to PS-MP followed by 90 days of depuration. Data (n = 10, per treatment) are presented as boxplots showing Median (horizontal line in boxplot), interquartile range, individual data points (in colour of box plots), and mean (red point). Asterisks (*) indicate statistically significant differences between control and treatment groups (* *p* < 0.05; ** *p* < 0.01; *** *p* < 0.001).

Oxidative stress marker enzymes (CAT, POD, and SOD) were analysed in the liver. No significant differences were found in CAT enzyme activity between the control and treatment groups (C1 vs. T1 − *P* = 0.393; C2 vs. T2 − *P* = 0.138) (Figure 2A). At day 21 of exposure, POD activity in the control group was significantly higher compared to the treatment group (*p* = 0.028). In contrast, an opposite but non-significant trend (*p* = 0.147) was observed after 90 days of depuration (Figure 2B). Regarding SOD, there were no significant differences in enzyme activity at day 21 post-exposure (*p* = 0.639). The second sampling for SOD was not analysed due to the loss of sample integrity (Figure 2C).

### 3.4. MDA Oxidative Damage Marker

**Figure 3:**
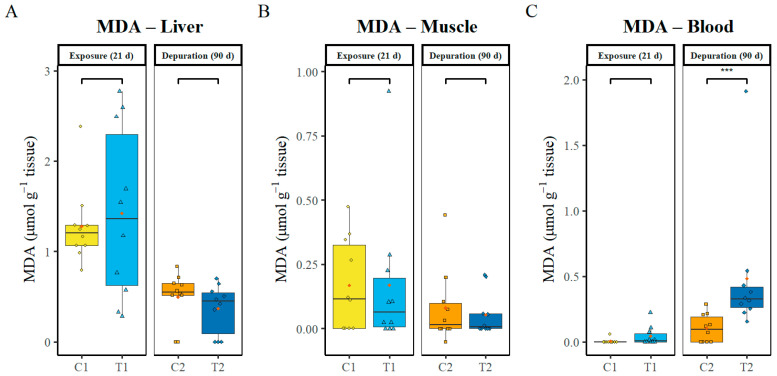
Oxidative stress marker MDA in liver (**A**), muscle (**B**) and blood (**C**). C1—control after 21 days, C2—control after 111 days, T1—treatment after 21 days of exposure to PS-MP, T2 treatment after 21 days of exposure to PS-MP followed by 90 days of depuration. Data (n = 10, per treatment) are presented as boxplots showing Median (horizontal line in boxplot), interquartile range, individual data points (in colour of box plots), and mean (red point). The brackets indicate Wilcoxon pairwise comparison, and asterisks (*) indicate statistically significant differences between control and treatment groups (* *p* < 0.05; ** *p* < 0.01; *** *p* < 0.001).

The oxidative damage marker malondialdehyde (MDA) was measured at two sampling points (C1 vs. T1, C2 vs. T2) in the liver, muscle, and blood (n = 10 per treatment). No significant differences in MDA levels were detected in the liver and muscle between the control and treatment groups at either sampling point (*p* ≥ 0.05) (Figure 3A,B). Regarding MDA concentrations in the blood, no significant difference between the control and treatment groups was found at day 21 of PS-MPs exposure (*p* = 0.81). At day 90, MDA levels were significantly higher (*p* = 0.03) in the treatment group (C2 vs. T2), indicating a persistent effect of MPs in this tissue (Figure 3C).

### 3.5. Digestion Enzyme Activity

**Figure 4:**
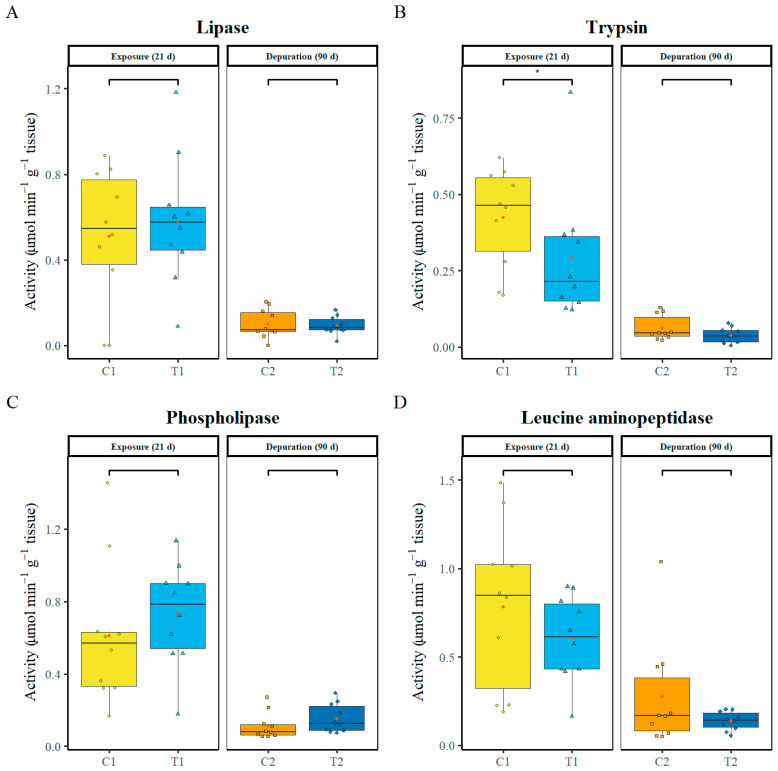
Digestion enzymes in the intestine: Lipase (**A**), Trypsin (**B**), phospholipase (**C**), and leucine aminopeptidase (**D**). C1—control after 21 days, C2—control after 111 days, T1—treatment after 21 days of exposure to PS-MP, T2 treatment after 21 days of exposure to PS-MP, followed by 90 days of depuration. Data are presented as boxplots showing Median (horizontal line in boxplot), interquartile range, individual data points (in colour of box plots), and mean (red point). Asterisks (*) indicate statistically significant differences between control and treatment groups (*p* < 0.05).

Enzymes related to protein and lipid digestion were assayed in intestinal tissue after 21 days of exposure to PS-MPs and following 90 days of depuration. Lipase showed no significant differences (C1 vs. T1 − *P* = 0.633; C2 vs. T2 − *P* = 0.905) in activity at the two sampling points (Figure 4A). Trypsin, on the other hand, showed a significant decrease after 21 days of PS-MP exposure (*p* = 0.043), whilst a subtle statistically not significant decrease was observed following 90 days of depuration (*p* = 0.247) (Figure 4B). Regarding phospholipase, at both sampling points no significant differences in enzyme activities were found (C1 vs. T1 − *P* = 0.315; C2 vs. T2 − *P* = 0.104) (Figure 4C). No significant differences were also observed in leucine aminopeptidase activity (C1 vs. T1 − *P* = 0.315; C2 vs. T2 − *P* = 0.684) (Figure 4D).

### 3.6. Metabolism Enzyme Activity

**Figure 5:**
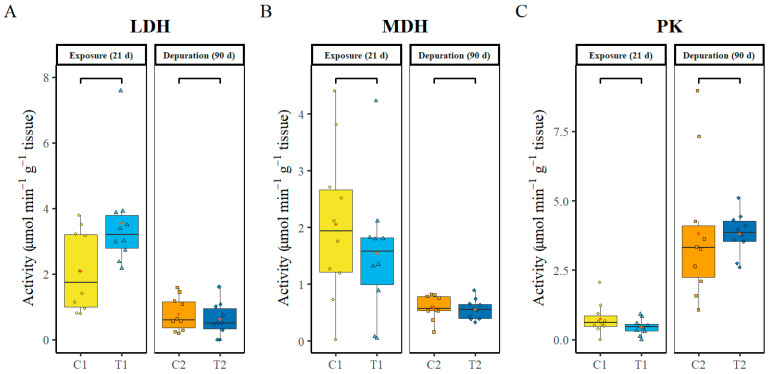
Metabolic enzymes in the liver: Lactate dehydrogenase (LDH) (**A**), malate dehydrogenase (MDH) (**B**), and pyruvate Kinase (**C**). C1—control after 21 days, C2—control after 111 days, T1—treatment after 21 days of exposure to PS-MP, T2 treatment after 21 days of exposure to PS-MP, followed by 90 days of depuration. Data (n = 10, per treatment) are presented as boxplots showing Median (horizontal line in boxplot), interquartile range, individual data points (in colour of box plots), and mean (red point).

Metabolic enzymes (LDH, MDH, PK) were analysed in the liver at day 21 post-exposure to PS-MP and after 90 days of depuration. No significant differences (*p* ≥ 0.05) were observed between the controls and treatment groups at each sampling time for all the metabolic enzymes (Figure 5A–C). It is interesting to note that LDH and MDH activities showed a decreasing trend from the first to the second sampling, while pyruvate kinase activity exhibited an opposite trend.

### 3.7. Inflammation Enzyme Activity

**Figure 6:**
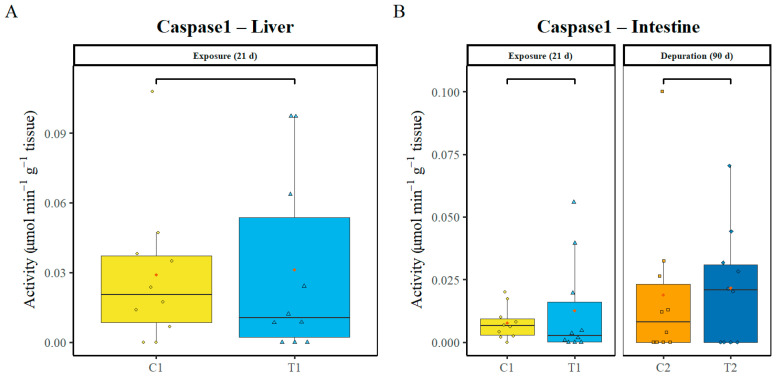
Inflammatory enzyme caspase-1 in the liver (**A**) and the intestines (**B**). C1—control after 21 days, C2—control after 111 days, T1—treatment after 21 days of exposure to PS-MP, T2 treatment after 21 days of exposure to PS-MP followed by 90 days of depuration. Data (n = 10, per treatment) are presented as boxplots showing Median (horizontal line in boxplot), interquartile range, individual data points (in colour of box plots), and mean (red point).

Caspase-1, a key enzyme related to inflammatory processes, was analysed in the liver and intestine. For the liver, only the day 21 exposure was analysed, and no statistically significant differences were found between the control and treatment groups (*p* = 681). After 90 days of depuration, caspase enzyme activities could not be analysed due to the loss of sample quality (Figure 6A). Regarding the intestine, at both time points, no significant differences (*p* ≥ 0.05) were detected in enzyme activities (Figure 6B).

### 3.8. Multivariate Biochemical Response Patterns

Principal component analysis (PCA) of standardised biochemical response variables, including metabolic and antioxidant enzyme activities together with oxidative stress biomarkers, revealed considerable overlap among all treatment groups (C1, T1, C2, and T2), indicating broadly similar biochemical profiles across exposure and depuration phases. The first two principal components explained 68.7% of total variance (PC1: 36.9%, PC2: 31.8%), with PC1 primarily influenced by caspase-1, lipase, and trypsin, and PC2 driven by lipase, trypsin, and leucine aminopeptidase (Supplementary Figure S1). Despite these distinct loading patterns, which reflect natural correlations among enzymes, substantial overlap among groups indicated no treatment-related shifts in the multivariate biochemical profile.

**Figure 7:**
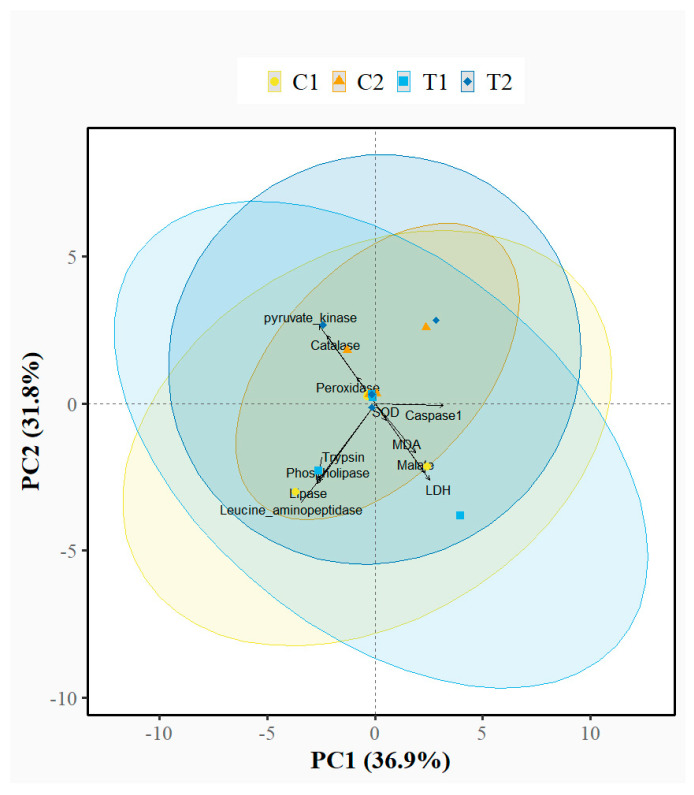
Principal component analysis (PCA) of standardised biochemical response variables, including enzyme activities and oxidative stress biomarkers of control and polystyrene-exposed fish. The ordination shows substantial overlap among control (C1, C2) and treatment (T1, T2) groups after 21 days and after 90 days, indicating broadly similar biochemical profiles. PERMANOVA revealed no significant effects of organ, time, or treatment (*p* > 0.05).

A two-way PERMANOVA using Euclidean distance and 999 permutations that incorporated organ (liver, intestine, muscle, blood), sampling time (21 days post exposure vs. 90 days depuration), and treatment group (control vs. treatment) explained 31.3% of the total variance but showed no significant effect of any factor or their interaction (F = 0.685, R^2^ = 0.313, *p* = 0.912) (Figure 7). These multivariate results are consistent with univariate Wilcoxon tests, which revealed significant differences in only a few selected parameters, specifically POD and trypsin activities and blood MDA concentrations following 90 days depuration, between control and PS-MP exposed fish during the exposure (C1 vs. T1) and depuration (C2 vs. T2) phases.

## 4. Discussion

This study investigated the tissue distribution of 1, 5, and 10 µm PS-MPs in the intestine, liver, and muscle of *Salmo trutta*, as well as their associated biochemical effects. A particle-number-based exposure approach was applied instead of the more commonly used mass-based methodology. This strategy was adopted because the effects of microplastic particles < 20 µm on fish have been largely neglected in both in vitro studies and environmental microplastic monitoring. The exposure level of approximately 5.4 × 10^6^ MPs g^−1^ feed represents a relatively high particle number; however, when expressed on a mass basis, it corresponds to only ~216.1 µg MP g^−1^ feed. This concentration falls within the range reported in environmental samples [34,35,36] and in various human tissues (5–500 µg g^−1^ tissue) [40,41,42,43,44]. Additionally, on a mass basis, the concentration used in the present study is comparable to those applied in previous studies [12,13,15,20,28]. Furthermore, the exposure design, comprising 21 days of dietary exposure followed by a 90-day depuration period, was intended to address critical knowledge gaps regarding the physiological effects of PS-MPs in *S. trutta* and their potential implications for aquaculture and food safety. Collectively, this integrated experimental approach has not been previously explored in microplastics research.

Microplastics concentration: PS-MP concentrations were quantified by particle size (1, 5, and 10 µm) in the liver, muscle, and intestine after 21 days of dietary exposure and following a 90-day depuration period. Only particles of 1 µm exhibited significant variation across tissues. This outcome is unsurprising, as smaller microplastics are known to display greater biological mobility [53]. In line with this, only 1 µm and 5 µm PS-MPs were detected in the liver at both sampling points, consistent with previous observations reported by Lu et al. [28] in *D. rerio*. However, it is worthwhile to note that other studies have reported larger MPs being detected in liver tissue, ranging from 1416 to 1634 µm in various species [9], 39–90 µm in European anchovy, 250 µm in fish from the Persian Gulf, and 214 ± 288 µm in gilt-head bream as cited in De Sales-Ribeiro et al. [54]. These findings are biologically questionable and may result from methodological or analytical errors, such as sample contamination, misidentification, or incomplete digestion of tissues.

With respect to retention, PS-MPs were detected in all examined organs even after 90 days of depuration. In the intestine, a significant reduction in 1 µm PS-MPs was observed during the depuration period, whereas no decrease was detected for 5 and 10 µm particles. This pattern may be explained by the greater ability of 1 µm particles to cross the intestinal barrier and enter systemic circulation, while larger particles are largely restricted to the gut lumen. This interpretation is supported by the consistently higher abundance of 10 µm PS-MPs in the intestine compared with the liver and muscle at both sampling time points. Larger particles (5 and 10 µm) may also be physically retained within intestinal folds. Consistent with this hypothesis, De Sales-Ribeiro et al. [54] reported that larger microplastics are more likely to be retained in the intestine than smaller particles. The same authors also note, however, that microplastic evacuation from the digestive tract can be rapid, highlighting the species- and size-dependent nature of retention dynamics. In contrast to this variable intestinal retention, accumulation in internal tissues showed a different pattern in the liver; concentrations of 1 and 5 µm PS-MPs were comparable at days 21 and 90. Similarly, PS-MP concentrations in muscle tissue did not differ significantly between sampling points, despite a non-significant, size-dependent increasing trend. Once PS-MPs cross the intestinal barrier and enter systemic circulation, a proportion is likely sequestered by primary filtering organs such as the liver, spleen, and kidney [20,55]. Particles that evade filtration may subsequently accumulate in peripheral tissues, including muscle. This dynamic distribution process likely underlies the observed accumulation patterns in liver and muscle and would be expected to continue until circulating microplastics are either retained by filtering organs or deposited in peripheral tissues. As a key result, the present study demonstrates that PS-MP concentrations in target organs did not decrease during the 90-day depuration period, indicating persistent retention rather than elimination.

Oxidative stress enzymes:

Since microplastics have previously been reported to induce widespread oxidative stress in fish tissues and organs [8,28], we assessed the effects of PS-MPs on oxidative stress–related enzymes, namely POD, SOD, and CAT, on day 21 immediately after PS-MP exposure and following 90 days of depuration. In the present study, only POD activity showed a significant decrease relative to the control after 21 days of exposure. In contrast, CAT and SOD activities did not differ significantly from control levels at either sampling point. Overall, the analysed oxidative stress–related enzymes provided no evidence of oxidative stress induction in the examined tissues.

Contradictory findings regarding the effects of microplastics on antioxidant enzyme activity have been reported in the literature. Kelly et al. (2024) [56] observed increased SOD, CAT, and glutathione peroxidase (GPx) activities in yellowtail kingfish (*Seriola lalandi*) following a two-day exposure to polyethene microplastics. Similarly, significant increases in CAT and SOD activities were reported in the gut and liver of zebrafish exposed to PS-MPs [28,57], and elevated antioxidant enzyme activities were also observed in *Coregonus peled* larvae following PS-MP exposure [58]. In contrast, decreased SOD, POD, and CAT activities were reported in the liver and muscle of *Nothobranchius guentheri* exposed to 5 and 15 µm PS-MPs [59]. Reduced antioxidant enzyme activities have also been documented in *Oreochromis niloticus* [60], larval *Danio rerio* [61], and larval *Cyprinus carpio* [62] following exposure to different types and concentrations of microplastics. Notably, in *C. carpio*, POD activity exhibited a dynamic response, with an initial increase followed by a decline during prolonged PVC microplastic exposure [62].

A detailed comparison of the cited studies reveals a general pattern: short-term, acute exposures (typically <96 h) frequently induce increases in antioxidant enzyme activity, likely reflecting an immediate adaptive defence response, whereas longer-term exposures (>7 days) tend to suppress these enzymes. This trend suggests that prolonged exposure to PS-MPs may overwhelm the antioxidant defence system, ultimately reducing its capacity to counteract oxidative stress. In this context, the findings of the present study are consistent with the existing literature.

Oxidative damage marker: Malondialdehyde (MDA) is a marker of lipid peroxidation and, therefore, an indicator of reactive oxygen species–induced damage. In the blood, MDA levels showed a clear trend of damage: they were nearly significant on day 21 (*p* = 0.06; Supplementary Table S1) and significantly increased after 90 days of depuration, indicating that oxidative damage was already developing at the earlier time point. These findings are supported by previous studies on *S. trutta*, which reported several adverse effects of microplastics on cellular and biochemical blood parameters, including decreases in erythrocyte and immune cell counts and reduced haemoglobin concentrations [19]. Similar changes in blood composition have also been observed in warm-water species, such as *C. catla* [18] (Rashid et al., 2024a) and *C. mrigala* [17] (Rashid et al., 2025). In contrast, no differences in MDA concentrations were detected in the blood of *Sparus aurata* exposed to low-density polyethene microplastics for 90 days, followed by a 30-day depuration period [63].

Overall, these results indicate a persistent damaging effect of PS-MP exposure on the circulatory system, a pattern that was also suggested in the liver, where MDA levels showed a near-significant increase (*p* = 0.09; Supplementary Table S1) following 90 days of depuration. Interestingly, MDA levels in muscle did not change, despite the observed increase in PS-MPs in muscle between day 21 and day 90 following depuration, suggesting that persistent MPs did not lead to measurable lipid peroxidation in this tissue.

Inflammatory responses: Caspase-1 is a key mediator of pyroptosis, a form of programmed cell death, and activates pro-inflammatory cytokines in response to cellular stress, including pollutants, pathogens, or environmental changes [64,65]. These signalling pathways are often triggered by oxidative stress [66,67]. In the present study, no significant differences in caspase-1 activity were detected in the liver or intestine of *S. trutta* at any sampling point, consistent with the lack of substantial oxidative stress in these organs. Previous studies report variable responses: in rats, only the highest dose of PS-MPs (1.5 mg/kg/day) increased caspase-1 expression, while lower doses had no effect [67]; in contrast, *Crucian carp* exposed to polyethene MPs for 21 days showed significant caspase-1 induction [68]. Our results suggest that, under the experimental conditions applied, the PS-MP concentration was insufficient to induce measurable inflammatory responses in either tissue.

Digestion Enzymes: In the present study, PS-MPs induced only selective biochemical responses in digestive enzymes. Among the digestive enzymes examined, only trypsin significantly decreased after 21 days of PS-MP exposure (Figure 4B), suggesting that PS-MP selectively impairs protein digestion. Our result is supported by Xiao et al. 2023 [59], who also found significant decreases in trypsin enzyme activities in *Nothobranchius guentheri* after 42 and 46 weeks of PS-MP exposure. The latter study also noted a significant decrease in other enzyme activities, including lipase, chymotrypsin and amylase. The present study results contrast with those reported for peled whitefish (*Coregonus peled*) exposed to PS microplastics at 5–500 µg L^−1^ for six days [58] and for silver barb (*Barbodes gonionotus*) fry exposed to PVC microplastics for 96 h [69]. These studies reported inconsistent changes in digestive enzyme activities, including chymotrypsin, carboxypeptidase, α-amylase, lipase, alkaline phosphatase, aminopeptidase, and esterases. Nevertheless, the observation of significant alterations in enzyme activity patterns across studies suggests that microplastic exposure may impair digestive tract function.

Metabolic Enzymes: Hepatic metabolic enzymes are commonly used as indicators of energy metabolism and cellular redox balance. In the present study, LDH, MDH, and PK activities showed no statistically significant differences between control and PS-MP-exposed groups at either sampling point, indicating no effect on hepatic metabolic function. Similarly, adult zebrafish exposed to 100–1000 µg L^−1^ polyethene and PS microplastics over 20 days showed no significant changes in LDH activity in whole-body homogenates [12]. In contrast, studies on *Oncorhynchus mykiss* and hybrid snakehead (*Channa* sp.) reported significant increases in LDH activity following chronic microplastic exposure [39,70], which might be interpreted as reflecting enhanced anaerobic glycolysis and redox compensation during metabolic stress [71]. The absence of significant changes indicates that PS-MP exposure under current experimental conditions did not induce a shift toward anaerobic energy metabolism. However, it is important to note that LDH activity was only marginally insignificant at the day 21 sampling (*p* = 0.007; all *p*-values are provided in Supplementary Table S1), suggesting that longer exposure or higher microplastic concentrations could have elicited significant increases.

MDH enzyme activity remained constant between sampling points, which is consistent with the findings of Morales-Espinoza et al. [72], who similarly observed no changes in *D. rerio* co-exposed to PET microplastics and cadmium for 21 days. In contrast, in the green chromide (*Etroplus suratensis*) muscle, a decrease in MDH activity was observed with increasing doses of PVC microplastics (1.02–10.78 mg L^−1^) after 42 days of exposure, indicating reduced ATP availability and impaired energy metabolism [73]. These contrasting results highlight the impact of polymer type, exposure duration, concentration, and tissue specificity on metabolic responses. Consistent with the patterns observed for LDH and MDH, PK activity also showed no significant shifts in the current study. This finding contradicts previous reports that polystyrene nanoparticles suppressed pyruvate kinase gene expression and reduced enzyme activity in channel catfish (*Ictalurus punctatus*) larvae [74] and Chinese horseshoe crab (*Tachypleus tridentatus*) [75]. The absence of PK inhibition adds to the evidence that PS-MP exposure had a minor impact on glycolytic energy production under the conditions tested.

Key Insights on Physiology, Aquaculture, and Ecology of Study Results:

The results of the present study provide essential insights into the physiological responses of *S. trutta* to PS-MPs. These data have relevance to aquaculture, ecology and future microplastic in vitro research. The study demonstrates that even high concentrations by numbers of PS-MPs exert only selective effects on key oxidative, digestive, and metabolic biomarkers in the liver and intestine of *S. trutta.* Principal component analysis supports this observation, showing no clear separation in enzyme activities or functional parameters related to oxidation, digestion, and metabolism between control and treatment groups across organs and sampling times. Although some shifts in enzyme activities were observed, most were not statistically significant, suggesting that under the present experimental conditions, high concentrations of PS-MPs induced only minor biochemical responses in *S. trutta*. Nonetheless, the observed tendencies imply that higher concentrations or longer exposure periods could result in more pronounced effects.

Given that *S. trutta* and related salmonids such as *O. mykiss* and *S. salar* are key aquaculture species, these findings have direct practical significance. Growth and survival, which are central to aquaculture performance, were unaffected throughout the experiment, and mortalities were less than 5%. However, these findings should be treated with caution, since the fish were not held under conditions reflective of standard aquaculture practices. Microplastics were detected in muscle tissue, which is particularly concerning since this tissue is consumed by humans. Remarkably, even after 90 days of depuration, microplastics persisted in the muscle without a decrease in concentration. This persistence indicates that once microplastics cross the intestinal barrier, they are difficult to eliminate, meaning that seemingly healthy fish may still contain micro- or nano-plastics in edible tissues. The results, therefore, emphasise that extended depuration periods, such as swimming in MP-free water, are insufficient for removing microplastics once they have translocated to the muscle. The most effective strategy to reduce microplastic accumulation is to prevent ingestion at the source.

This study also highlights potential seasonal variations in enzyme activities. Enzyme activities generally increased at the second sampling time point (90 days after depuration). This pattern was also observed in the control group, indicating that these shifts are seasonal. In many temperate-zone fish species, digestive processes, metabolism, and growth typically decline during winter (i.e., when the MP exposure started), likely reflecting an intrinsic biological rhythm that reduces metabolic activity as fish enter a hibernation-like state [76], and increase during spring conditions (i.e., at the end of the depuration period). These findings underscore the importance of considering temporal and seasonal factors when interpreting enzyme activity data in microplastic studies. Furthermore, in long-term experiments such as this one, comparisons across sampling times must account for natural seasonal fluctuations to avoid misattributing biological variation to experimental treatments.

As a limitation of this study, we examined the effects of multiple PS-MP size classes simultaneously. Future studies should focus on the size ranges that show the strongest effects to achieve higher experimental resolution and clearer results.

## 5. Conclusions

Our results show that PS-MPs of 1–10 µm translocate from the intestine to liver and muscle in *S. trutta*, with the smallest particles (1 µm) displaying the greatest mobility and accumulation across all tissues. This supports the initial hypothesis of size-dependent translocation. Regarding biochemical effects, despite exposure to relatively high particle numbers, only selective and transient responses were observed, such as reduced POD and trypsin activities on day 21 post-exposure. Oxidative damage in the blood appeared delayed, and inflammatory responses were unaffected. Consequently, the initial hypothesis that this exposure would provoke a strong biochemical response was only partially supported.

For translocation and persistence, the hypothesis was a consistent, drastic reduction in all organs after depuration. This was only confirmed in the intestine. In the muscle and liver, PS-MP levels did not change significantly after 90 days of depuration. Overall, PS-MP translocation and persistence depend on particle size and the specific organ. Finally, this study highlights discrepancies in ecotoxicological exposure methodologies. It demonstrates that high particle numbers of small microplastics (≤10 µm) are not inherently environmentally unrealistic and will not always induce widespread toxicity in commonly assessed biochemical markers. Therefore, exposure methodologies should report particle number, size, and shape to enable proper comparison between particle- and mass-based studies. In this pilot study, the effects of multiple PS-MP size classes were assessed collectively. The findings suggest that particle numbers used in some mass-based studies may be unrealistically high to elicit the reported effects. To enable accurate risk assessment, future environmental monitoring should place greater emphasis on quantifying microplastic particle numbers across all relevant size classes, particularly the smaller sizes, which are more biologically mobile. Likewise, future experimental studies should focus on the size class with the highest biological mobility to improve the resolution and interpretability of results.

## Figures and Tables

**Table 1:** Microplastic dilution, feed incorporation and verification in feed.

Particle Size ^1^	PS-MP Concentration in Stock Suspension (Particles/mL) ^1^	PS-MPs Added to 3 kg Feed Batch ^2^	Analytically Recovered PS ^2^ (Particles/g Feed)	Calculated PS Mass ^2^ (µg/g Feed)
1 µm	3.656 × 10^11^	2.0 × 10^10^	4.2 × 10^6^	2.3
5 µm	1.4624 × 10^9^	2.0 × 10^10^	4.2 × 10^5^	28.8
10 µm	1.828 × 10^8^	2.0 × 10^10^	4.2 × 10^5^	230.5

^1^ Particle diameter and concentrations are reported as declared by the manufacturer’s product certificate, Sigma-Aldrich. ^2^ All PS-MP numbers and masses are referred to the dry feed mass.

**Table 2:** Recovered polystyrene microplastics particles × 10^6^ after 21 days of exposure and following 90 days of depuration.

Organ	Particle Size	Exposure (21 d) Mean ± SD	Depuration (90 d) Mean ± SD	Percent Difference	*p*-Value
Liver	1 µm	0.768 ± 1.088	0.551 ± 1.159	−28.32 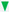 ^1^	0.132
	5 µm	0.169 ± 0.513	0.355 ± 0.852	109.51 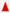 ^2^	0.233
	10 µm	Non detected	Non detected		
Muscle	1 µm	0.145 ± 0.234	0.210 ± 0.311	44.57 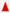	0.757
	5 µm	0.043 ± 0.090	0.043 ± 0.083	−1.243 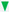	0.914
	10 µm	0.0055 ± 0.023	0.0126 ± 0.0461	130.0 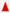	0.647
Intestine	1 µm	0.291 ± 0.546	0.081 ± 0.2101	−72.18 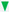	0.013 *
	5 µm	0.218 ± 1.039	0.0327 ± 0.109	−84.96 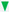	0.328
	10 µm	0.0786 ± 0.284	0.0142 ± 0.064	−81.94 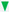	0.413

Green triangles indicate a decrease ^1^, whereas red triangles indicate an increase in PS-MP abundance after depuration ^2^. Asterisks (*) indicate statistically significant differences between recovered polystyrene microplastics after 21 days of exposure and following 90 days of depuration (* *p* < 0.05).

## Data Availability

All data generated in this research can be made available upon request.

## Supplementary Materials

The following supporting information can be downloaded at: https://www.mdpi.com/article/10.3390/ani16020285/s1, Figure S1: Variable contribution to PCA dimension; Table S1: Enzyme Activity and MDA concentration in the analysed organs; Table S2: Foreign microplastic quantification.
