# Supplementary material for: Size-Dependent Tissue Translocation and Physiological Responses to Dietary Polystyrene Microplastics in Salmo trutta
Source: Animals (Basel). 2026 Jan 16;16(2):285. doi: 10.3390/ani16020285 (PMC12837546; doi:10.3390/ani16020285)
Supplement: Supplementary file 1 [file animals-16-00285-s001.zip › animals-4048692-supplementary.pdf]

Supplementary file

Table S1. Enzyme Activity and MDA concentration in the analysed organs.

Enzyme	Organ	Sampling Time	Treatment	n	Mean ± SD	P-value	P-label
Caspase1	Intestine	Depuration (90 d)	Control	10	0.02 ± 0.03	0.668	ns
Caspase1	Intestine	Depuration (90 d)	Treatment	10	0.02 ± 0.02	0.668	ns
Caspase1	Intestine	Exposure (21 d)	Control	10	0.01 ± 0.01	0.448	ns
Caspase1	Intestine	Exposure (21 d)	Treatment	10	0.01 ± 0.02	0.448	ns
Caspase1	Liver	Exposure (21 d)	Control	10	0.03 ± 0.03	0.732	ns
Caspase1	Liver	Exposure (21 d)	Treatment	10	0.03 ± 0.04	0.732	ns
Catalase	Liver	Depuration (90 d)	Control	10	84.33 ± 98.12	0.231	ns
Catalase	Liver	Depuration (90 d)	Treatment	10	214.77 ± 262.81	0.231	ns
Catalase	Liver	Exposure (21 d)	Control	10	31.91 ± 15.92	0.393	ns
Catalase	Liver	Exposure (21 d)	Treatment	10	38.84 ± 23.49	0.393	ns
LDH	Liver	Depuration (90 d)	Control	10	0.78 ± 0.51	0.52	ns
LDH	Liver	Depuration (90 d)	Treatment	10	0.62 ± 0.50	0.52	ns
LDH	Liver	Exposure (21 d)	Control	10	2.08 ± 1.29	0.077	ns
LDH	Liver	Exposure (21 d)	Treatment	10	3.62 ± 1.61	0.077	ns
LAP	Intestine	Depuration (90 d)	Control	10	0.28 ± 0.31	0.684	ns
LAP	Intestine	Depuration (90 d)	Treatment	10	0.14 ± 0.05	0.684	ns
LAP	Intestine	Exposure (21 d)	Control	10	0.78 ± 0.47	0.315	ns
LAP	Intestine	Exposure (21 d)	Treatment	10	0.60 ± 0.24	0.315	ns
Lipase	Intestine	Depuration (90 d)	Control	10	0.10 ± 0.07	0.853	ns
Lipase	Intestine	Depuration (90 d)	Treatment	10	0.09 ± 0.04	0.853	ns
Lipase	Intestine	Exposure (21 d)	Control	10	0.57 ± 0.28	0.968	ns
Lipase	Intestine	Exposure (21 d)	Treatment	10	0.58 ± 0.30	0.968	ns
MDA	Blood	Depuration (90 d)	Control	10	0.17 ± 0.08	0.0319	*
MDA	Blood	Depuration (90 d)	Treatment	10	0.28 ± 0.08	0.0319	*
MDA	Blood	Exposure (21 d)	Control	10	0.01 ± 0.02	0.0623	ns
MDA	Blood	Exposure (21 d)	Treatment	10	0.04 ± 0.07	0.0623	ns
MDA	Liver	Depuration (90 d)	Control	10	0.64 ± 0.11	0.0973	ns
MDA	Liver	Depuration (90 d)	Treatment	10	0.52 ± 0.12	0.0973	ns
MDA	Liver	Exposure (21 d)	Control	10	1.28 ± 0.43	0.853	ns
MDA	Liver	Exposure (21 d)	Treatment	10	1.42 ± 0.95	0.853	ns
MDA	Muscle	Depuration (90 d)	Control	10	0.17 ± 0.16	0.931	ns
MDA	Muscle	Depuration (90 d)	Treatment	10	0.12 ± 0.09	0.931	ns
MDA	Muscle	Exposure (21 d)	Control	10	0.17 ± 0.18	0.728	ns
MDA	Muscle	Exposure (21 d)	Treatment	10	0.17 ± 0.28	0.728	ns
MDH	Liver	Depuration (90 d)	Control	10	0.59 ± 0.22	0.684	ns
MDH	Liver	Depuration (90 d)	Treatment	10	0.56 ± 0.18	0.684	ns
MDH	Liver	Exposure (21 d)	Control	10	2.05 ± 1.36	0.579	ns
MDH	Liver	Exposure (21 d)	Treatment	10	1.55 ± 1.19	0.579	ns
POD	Liver	Depuration (90 d)	Control	10	0.95 ± 0.88	0.773	ns
POD	Liver	Depuration (90 d)	Treatment	10	1.09 ± 1.35	0.773	ns
POD	Liver	Exposure (21 d)	Control	10	1.57 ± 1.01	0.0021	**
POD	Liver	Exposure (21 d)	Treatment	10	0.28 ± 0.34	0.0021	**
Phospholipase	Intestine	Depuration (90 d)	Control	10	0.11 ± 0.07	0.104	ns
Phospholipase	Intestine	Depuration (90 d)	Treatment	10	0.15 ± 0.08	0.104	ns
Phospholipase	Intestine	Exposure (21 d)	Control	10	0.61 ± 0.39	0.315	ns

Phospholipase	Intestine	Exposure (21 d)	Treatment	10	0.73 ± 0.28	0.315	ns
SOD	Liver	Exposure (21 d)	Control	10	0.81 ± 1.08	0.31	ns
SOD	Liver	Exposure (21 d)	Treatment	10	2.71 ± 4.40	0.31	ns
Trypsin	Intestine	Depuration (90 d)	Control	10	0.06 ± 0.04	0.247	ns
Trypsin	Intestine	Depuration (90 d)	Treatment	10	0.04 ± 0.03	0.247	ns
Trypsin	Intestine	Exposure (21 d)	Control	10	0.43 ± 0.16	0.0433	*
Trypsin	Intestine	Exposure (21 d)	Treatment	10	0.29 ± 0.22	0.0433	*
Pyruvate kinase	Liver	Depuration (90 d)	Control	10	3.82 ± 2.50	0.315	ns
Pyruvate kinase	Liver	Depuration (90 d)	Treatment	10	3.81 ± 0.76	0.315	ns
Pyruvate kinase	Liver	Exposure (21 d)	Control	10	0.74 ± 0.57	0.173	ns
Pyruvate kinase	Liver	Exposure (21 d)	Treatment	10	0.45 ± 0.29	0.173	ns

Table S1. Summary of enzyme activities and malondialdehyde (MDA) concentrations. Enzyme activities are expressed as mean ± SD (μmol/min/g tissue), and MDA concentrations as mean ± SD (μmol/g tissue). Pairwise comparisons between treatments were performed for each organ and sampling time using the Wilcoxon rank-sum test (*$p < 0.05$; **$p < 0.01$; ***$p < 0.001$; ns = not significant). Blue-shaded rows indicate statistically significant differences, and green-shaded rows indicate trends toward significance ($0.05 \leq p < 0.1$). Abbreviations: LDH, Lactate Dehydrogenase; MDH, Malate Dehydrogenase; POD, Peroxidase; MDA, Malondialdehyde.

Table S2. Foreign microplastic quantification

Microplastic particle concentrations varied across tissues, with mean values (± SD) of 373,300 ± 276,400 particles g^{-1} in the intestine, 1,150,400 ± 1,048,400 particles g^{-1} in the liver, and 1,180,100 ± 787,200 particles g^{-1} in muscle. To estimate the mass of foreign microplastics (MPs), the volume of each particle was multiplied by 1.14 g/cm^3. This value represents the average specific density of common microplastics found in aquatic environments.
Foreign microplastics were predominantly non-spherical and larger than the reference polystyrene (PS) microspheres (0.8–1.7 μm diameter). They displayed varied morphologies, including elongated and irregular shapes (length: 0.62–2.72 μm; width: 1.00–1.18 μm). Occasionally, large fibres were observed but excluded as potential contamination artefacts.

All particle types detected concentration			Particle type		
**Organ**	**Particle concentration (particles/g)**	**Particle concentration (ng/g)**	**Approximately spherical Particle diameter (μm)**	**Fibre Particle length (μm)**	**Fibre Particle diameter (μm)**
Intestine	373,318.33 ± 276,400	0.927	0.799 ± 0.122	0.626 ± 0.090	1.001 ± 0.120
Liver	1,150,427 ± 1,048,400	16.744	1.416 ± 0.118	0.00	0.00
Muscle	1,180,100 ± 787,200	27.613	1.730 ± 0.244	0.224	0.212

Table S2. Concentrations and physical characteristics of foreign microplastics (MPs) detected in fish tissues. Values represent mean particle concentration (particles/g wet tissue ± SD), estimated mass concentration (ng/g), and the dimensions of the predominant particle morphologies (approximately spherical particles and fibres.
****Note:** Corresponding particle images are presented in Fig. 1 of the main document.

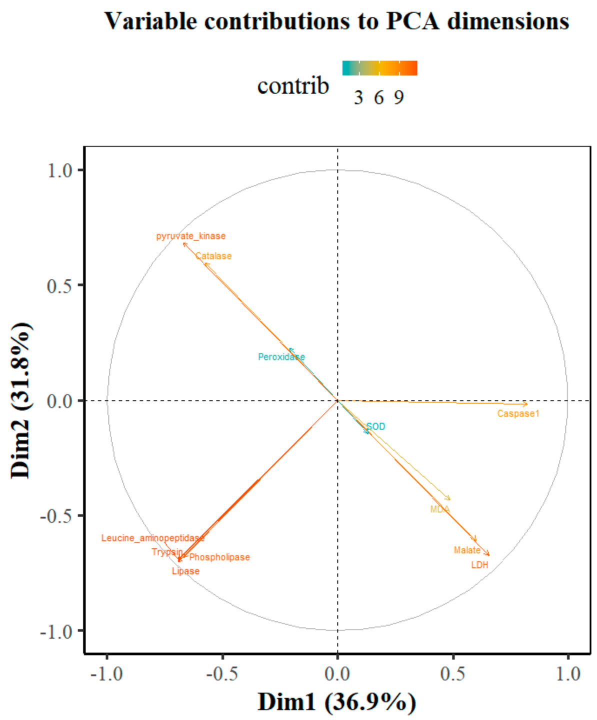

Figure S1. Variable contribution to PCA dimension, the first two principal components explained 68.7% of total variance (PC1: 36.9%, PC2: 31.8%), with PC1 primarily influenced by Caspase-1, Lipase, and Trypsin, and PC2 driven by Lipase, Trypsin, and Leucine aminopeptidase.
